# Inflammatory PAF Receptor Signaling Initiates Hedgehog Signaling and Kidney Fibrogenesis During Ethanol Consumption

**DOI:** 10.1371/journal.pone.0145691

**Published:** 2015-12-31

**Authors:** Calivarathan Latchoumycandane, Mohamad Hanouneh, Laura E. Nagy, Thomas M. McIntyre

**Affiliations:** 1 Department of Cellular and Molecular Medicine, Lerner Research Institute, Cleveland Clinic Lerner College of Medicine, Cleveland, Ohio, United States of America; 2 Department of Internal Medicine, Cleveland Clinic, Cleveland, Ohio, United States of America; 3 Department of Pathobiology, Lerner Research Institute, Cleveland Clinic Lerner College of Medicine, Cleveland, Ohio, United States of America; National Cancer Institute, UNITED STATES

## Abstract

Acute inflammation either resolves or proceeds to fibrotic repair that replaces functional tissue. Pro-fibrotic hedgehog signaling and induction of its Gli transcription factor in pericytes induces fibrosis in kidney, but molecular instructions connecting inflammation to fibrosis are opaque. We show acute kidney inflammation resulting from chronic ingestion of the common xenobiotic ethanol initiates Gli1 transcription and hedgehog synthesis in kidney pericytes, and promotes renal fibrosis. Ethanol ingestion stimulated transcription of TGF-ß, collagens I and IV, and alpha-smooth muscle actin with accumulation of these proteins. This was accompanied by deposition of extracellular fibrils. Ethanol catabolism by CYP2E1 in kidney generates local reactive oxygen species that oxidize cellular phospholipids to phospholipid products that activate the Platelet-activating Factor receptor (PTAFR) for inflammatory phospholipids. Genetically deleting this *ptafr* locus abolished accumulation of mRNA for TGF-ß, collagen IV, and α-smooth muscle actin. Loss of PTAFR also abolished ethanol-stimulated Sonic (Shh) and Indian hedgehog (Ihh) expression, and abolished transcription and accumulation of Gli1. Shh induced in pericytes and Ihh in tubules escaped to urine of ethanol-fed mice. Neutrophil myeloperoxidase (MPO) is required for ethanol-induced kidney inflammation, and Shh was not present in kidney or urine of *mpo* ^-/-^ mice. Shh also was present in urine of patients with acute kidney injury, but not in normal individuals or those with fibrotic liver cirrhosis We conclude neither endogenous PTAFR signaling nor CYP2E1-generated radicals alone are sufficient to initiate hedgehog signaling, but instead PTAFR-dependent neutrophil infiltration with myeloperoxidase activation is necessary to initiate ethanol-induced fibrosis in kidney. We also show fibrogenic mediators escape to urine, defining a new class of urinary mechanistic biomarkers of fibrogenesis for an organ not commonly biopsied.

## Introduction

Ethanol is the most commonly ingested xenobiotic agent, and while its use is difficult to precisely quantitate, it is estimated that 8% of the American adult population regularly use alcohol [1]. Chronic ethanol ingestion would appear to have little discernable effect on kidney function since blood urea nitrogen and creatinine in the circulation that reflect renal filtration are not routinely elevated in this population, although these two assessments are known as delayed and insensitive markers of kidney function [2]. However, the sudden onset of acute kidney injury in patients hospitalized with severe alcoholic hepatitis very tightly associates with mortality [3]. This occurs for unknown reasons, and this rapid decompensation of kidney function suggests that there is, in fact, an at-risk population of individuals with alcohol use disorder that is undetectable by current tests.

Exogenous ethanol is metabolized by three pathways in liver, with CYP2E1 catabolism [4] generating reactive oxygen species (ROS)[5] that injures liver [6]. Kidney expresses about a tenth of the body’s CYP2E1 content [7,8], and so, like liver, kidney metabolizes circulating ethanol with local generation of damaging ROS [9]. Oxidants directly damage tissue, but also induce inflammation [10] and cell death [11] through the inflammatory phospholipid mediator Platelet-activating Factor (PAF). PAF stimulates platelet, but also neutrophil, function at sub-picomolar concentrations [12] through a single receptor (PTAFR) expressed by nearly all cells of the innate immune system [13]. An essential element of PTAFR recognition of the inflammatory phospholipid PAF is its short *sn*-2 acetyl residue [14]. However ROS attack on cellular phospholipids can truncate their esterified polyunsaturated acyl residues, forming structural analogs of PAF with short *sn*-2 residues [15]. Some of these oxidation products also stimulate PTAFR [16], and so are biologically active [11,17–19]. The difference between biosynthetic PAF production and oxidatively truncated phospholipids is that ROS formation produces inflammatory and apoptotic mediators through chemical reactions that are not subject to biologic control.

Sonic hedgehog (Shh) and Indian hedgehog (Ihh) are pro-fibrotic agonists in a variety of tissues [20–23] that act by binding to the receptor Patched (*Ptch*). This ligation relieves Patched inhibition of Smoothened (*Smo*) that then activates the transcription factor Gli1. This induces Shh production in a positive feedback loop in differentiated kidney pericytes [24–27] subsequent to ischemic kidney injury [22]. However, the initial stimulus that connects inflammation to autocrine hedgehog activation remains occult.

Chronic ingestion and catabolism of ethanol in the pre-clinical Lieber-deCarli model initiates a neutrophilic inflammatory infiltrate in kidney that severely damages filtration and causes the syndrome of acute kidney injury [10,28]. Genetic ablation of PTAFR blocks this renal inflammation and fully protects the kidney from ethanol-induced inflammatory damage, as does deletion of myeloperoxidase [28], the oxidative, bactericidal enzyme of activated neutrophils.

Here, we show chronic ethanol ingestion induced collagen mRNA and protein accumulation in kidney with extracellular fibril deposition, and that this initiation of fibrosis requires functional PTAFR and myeloperoxidase. Ethanol ingestion induced the hedgehog pathway in kidney with Ihh expression in kidney tubules, where CYP2E1 is expressed. Ethanol ingestion also stimulated Shh accumulation in kidney pericytes that are the fibrotic cells of kidney [27,29]. These two hedgehog mediators escaped to urine, and so uniquely mark on-going fibrosis in an organ not generally subject to biopsy. Shh was significantly elevated in the urine of human patients with acute kidney injury that was independent of cirrhotic liver fibrosis. Urinary Shh thus correlates to kidney, and not liver, injury.

We conclude chronic ethanol metabolism alone is sufficient to initiate renal fibrosis and hedgehog production, and that urinary hedgehogs report on-going fibrosis within kidney. We conclude local ROS generated during ethanol catabolism is insufficient to provoke the fibrotic response, but instead requires autocrine PTAFR signaling of neutrophilic infiltration and activation. Finally, we conclude that similar fibrotic repair is likely to occur in the kidneys of humans who develop acute kidney injury since this patient population also sheds Shh to urine.

## Material and Methods

### Materials

Antibodies to TGF-ß-1, collagen IV, collagen 1, Gli1 and Indian hedgehog were from Abcam (Cambridge, MA, USA). Antibody to PGDFR-ß1 was from Cell Signaling Technology (Danvers, MA). Sonic hedgehog and alpha-smooth muscle actin antibodies, ß-actin, the ß-galactosidase reporter gene staining kit and all other reagents were supplied by Sigma-Aldrich (St. Louis, MO, USA).

### Animal husbandry

Animals received humane care in a protocol approved by the Cleveland Clinic Institutional Animal Care and Use Committee that conforms to the *Guide for the Care and Use of Laboratory Animals* (NIH Publication No. 85–23, revised 1996). Adult male Wistar rats (~170–180 g) were purchase from Harlan Sprague Dawley (Indianapolis, IN, USA). Female C57BL/6J mice from Jackson Labs (Bar Harbor, Maine). PAFR null mice were the generous gift of Takao Shimizu (University of Tokyo) after back breeding to the BL6 strain (Jeffery Travers, Indiana University). *mpo* ^*-/-*^ and BL6 mice were the gift of Stanley Hazen (Cleveland Clinic). Lieber-deCarli ethanol diets were from Dyets (Bethlehem, PA). Gli1^tm2Alj^/J with β-galactosidase knocked in to the *gli1* locus were from the Jackson Laboratory and were used as heterozygotic reporter mice.

The chronic ethanol-feeding model used in this study has been previously described [30]. Briefly, age-matched rats were randomly assigned to be ethanol fed or pair fed a control diet in which maltose dextrin isocalorically substituted for ethanol in the liquid diet to maintain equal body weights. For the first 2 days of the protocol, rats in the ethanol group were fed a liquid diet with 17% of the calories supplied as ethanol and then were provided an ad libitum liquid diet for 4 weeks containing 6.4% (v/v) ethanol, which constitutes 36% of the total caloric content. For murine experiments, mice (8–10 weeks old) were randomly assigned to the ethanol or control arm of the protocol. Control mice were fed with maltose dextrin that was isocalorically substituted for ethanol based on the caloric intake of the ethanol-fed mice on the previous day. This pair feeding insures the weight gains during the trial were equal. Ethanol-fed mice were allowed free access to an ethanol containing liquid diet where the concentration of ethanol was ramped over time starting from 1% and 2% (vol/vol) each for 2 days, then 4% and 5% ethanol each for 7 days, and finally 6% ethanol for 1 week. The 6% (vol/vol) ethanol diet provides 32% of total calories in the diet. After ethanol or pair feeding, the rats or mice were anesthetized by CO_2_, exsanguinated, and serum isolated and stored at −80°C. Kidney tissues were isolated and stored in RNAlater^TM^ for RNA quantification, urine was collected by bladder puncture after euthanasia, and remaining tissues were stored at -80°C for protein and lipid determination. A small portion of the kidney tissues was immediately fixed in 10% formalin for histology.

### Patient cohorts

This study was conducted according to the principles expressed in the Declaration of Helsinki. De-identified urine samples were collected with approval of Cleveland Clinic’s Institutional Review Board (15–261, “Urinary biomarkers to differentiate between acute tubular necrosis and hepatorenal syndrome in patients with liver cirrhosis.”) Written informed consent, using a form specifically approved by the Cleveland Clinic IRB for this study, was obtained in a private setting in the patient's room at the clinic or hospital during clinic visits or during hospital stays. The consent was obtained by the author Mohammad Hanouneh, MD, with patients allowed sufficient time to study the consent form before being recruited into the study. These patients from the Internal Medicine department of the Cleveland Clinic had been diagnosed with acute kidney injury, acute kidney injury and cirrhosis (S1 Table), or cirrhosis without acute kidney injury. The diagnosis of liver cirrhosis was based on the histological features of cirrhosis on liver biopsy and/or a composite of clinical signs and findings of cirrhosis provided by laboratory tests, endoscopy, and radiologic imaging. Acute kidney injury was defined as abrupt (less than 48 hours) reduction in kidney function manifested by an increase in serum creatinine of more than 0.3 mg/dL from the baseline or by an increase in serum creatinine of 50% or more. Samples (25 μL) of urine were directly resolved with 6x sample buffer by SDS-PAGE, and western blotted for Shh.

### Western blotting

Urine samples from rats or mice were collected by bladder puncture, mixed with 6x Laemmli buffer containing 10% SDS and 200 mM DDT, followed by boiling. SDS-PAGE used 10 to 12% gels that were blotted onto nitrocellulose membranes (Bio-Rad) and blocked with 5% nonfat dry milk (Bio-Rad). Detection used anti-sonic hedgehog (Sigma, 1:3000) and anti-Indian hedgehog (Abcam, 1:2000) antibodies incubated overnight at 4°C. The conjugates were then ligated by horseradish peroxidase-conjugated anti-mouse (1:10000) or anti-rabbit (1:5000) antibody before detection with Amersham Biosciences ECL Prime. Gels contained equal volumes of urine to allow comparison among lanes since urine contains variable amounts of protein so that loading controls are not possible. Kidney tissues from ethanol-fed and pair-fed control mice were washed with ice cold PBS and homogenized on ice in a Potter-Elvehjem glass homogenizer containing 1x lysis buffer (Cell Signaling Technology) with protease inhibitor (Sigma, MO). The homogenates were cleared by centrifugation for 30 min at 14,000 x *g*. For western blotting, samples were mixed with 6x Laemmli buffer containing 10% SDS and 200 mM DTT, followed by boiling. SDS-PAGE used 10–12% gels that were blotted onto nitrocellulose membranes (Bio-Rad). Detection used anti-Indian hedgehog (Abcam, 1:2000) or Collagen 1 (Abcam, 1:3000) or **β**-Actin (loading control) antibodies incubated overnight at 4°C. The conjugates were then ligated by horseradish peroxidase-conjugated anti-mouse (1:10000) or anti-rabbit (1:5000) antibody before detection with Amersham Biosciences ECL Prime.

### RNA Isolation and qPCR

Total RNA was extracted from kidney tissues preserved in RNAlater (Qiagen, Germantown, MD) using RNeasy mini kits (Qiagen, Germantown, MD). RNA content was measured in a NanoDrop ND-1000 spectrophotometer. Messenger RNA was quantified by SYBR Green one-step reverse transcription-PCR for TGF-ß-1, **α**-SMA and Collagen IV, Sonic hedgehog, Gli1 and 18S with the Bio-Rad MyiQ real-time PCR detection system. The primers for rat TGFβ-1 (sense, 5’-TGA GTG GCT GTC TTT TGA CG-3’; antisense, 5’-GTT TGG GAC TGA TCC CAT TG-3’), alpha-smooth muscle actin (sense, 5’- GAT CAC CAT CGG GAA TGA AC-3’; antisense, 5’-ATA GGT GGT TTC GTG GAT GC-3’), collagen type IVa1 (sense, 5’-AAC CTG GCA GTG ATG GAA TC-3’; antisense, 5’-TCA CCC TTG GAA CCT TTG TC-3’) and the primers for mouse TGF-ß-1 (sense, 5’-TGA GTG GCT GTC TTT TGA CG-3’; antisense, 5’-AGT GAG CGC TGA ATC GAA AG-3’), alpha−smooth muscle actin (sense, 5’- TTG CTG ACA GGA TGC AGA AG-3’; antisense, 5’-TGA TCC ACA TCT GCT GGA AG-3’) mouse collagen IV (sense, 5’-AAA GGG AGA AAG AGG CTT GC-3’; antisense, 5’-AGC ATC ACC CTT TTG TCC TG-3’), mouse collagen 1 a1 (sense, 5’ GGC AAG AAT GGA GAT GAT GG-3’; antisense, 5’-ACC ATC CAA ACC ACT GAA GC-3’), mouse Sonic-hedgehog (sense, 5’-CCA ATT ACA ACC CCG ACA TC-3’; antisense, 5’-TCA TCA CAG AGA TGG CCA AG-3’) and mouse Gli1 (sense, 5’-TGT GTG AGC AAG AAG GTT GC-3’; antisense, 5’-ATG GCT TCT CAT TGG AGT GG-3’) were purchased from IDT (Coralville, IA). The mRNA expression was normalized to 18S mRNA content and 2^-∆∆CT^ was used to calculate the fold change.

### TGF-ß-1 Immunohistochemistry

Rat kidneys were fixed in 10% buffered formalin, embedded in paraffin, and sectioned (Histology Core, Cleveland Clinic). Paraffin embedded sections were deparaffinized in Safeclear II xylene substitute and then consecutively hydrated in 100, 95, 85 and 70% ethanol followed by two washes in PBS. The sections were treated with 10 mM citrate buffer (pH 6.0) at 95–100°C for 15 min for antigen retrieval, incubated with peroxidase block (Pierce) for 30 min, washed and blocked with 10% donkey serum before incubation with polyclonal anti-rabbit TGF-ß-1 (Abcam, 1:200). After three washes in PBS, the sections were incubated with SP-conjugated streptavidin for 30 min followed by HRP for 30 min. After washing in PBS, the sections were treated with substrate-chromogen solution (diaminobenzoate/ metal concentrate; Pierce) for 2–5 min at room temperature before the sections were washed and mounted. Images were acquired with a 60x objective.

### Immunofluorescence

After deparaffinization and hydration, kidney sections were treated with proteinase K (20 ug/ ml, 15 min) for antigen retrieval. The sections were washed and blocked with 10% donkey serum and 0.1% Triton X-100 in phosphate buffered saline and incubated overnight with antibodies to alpha-smooth muscle actin (1:200), collagen type IV (1:100), sonic hedgehog (1:200), Gli1 (1:200) or Indian hedgehog (1:50). Washed sections were incubated with 1:1000 Alexa Fluor488 or Alexa Fluor568-conjugated donkey anti-rabbit or anti-mouse (Invitrogen) antibody. Sections were washed and mounted with DAPI-containing Vectashield (Vector Laboratories). Images were acquired using a 60x objective.

### ß-Galactosidase Reporter Gene Staining

Frozen kidney sections of LacZ reporter mice (Gli1^tm2Alj^/J with ß-galactosidase knocked in to a single *gli1* locus) were stained for ß-galactosidase expression using β-galactosidase Reporter Gene Staining Kit (Sigma) strictly according to the manufacturer’s protocol. Briefly, frozen kidney sections were fixed (2% formaldehyde and 0.2% glutaraldehyde) for 20 min and then stained with 1 ml of staining solution (MgCl_2_, potassium ferricyanide, potassium ferrocyanide, and 5-Bromo-4-chloro-3-indolyl-ß-D-galactopyranoside in PBS) at 37°C for 2 hr. After mounting, the slides were observed under a 60x objective.

### Transmission Electron Microscopy

Kidney tissues from rats and mice were cut into small slices that were then fixed in 2.0% paraformaldehyde and 2.5% glutaraldehyde (both E.M. grade) in 0.1 M Na-Cacodylate buffer, pH 7.4. Kidney sections were further processed and analyzed through the Imaging Core of the Cleveland Clinic.

### Statistical Analysis

All data are presented as mean ± S.E. Independent Student’s t test (two groups) or one-way analysis of variance (multiple groups). These tests were performed by GraphPad Prism5 statistics software. Statistical significance was considered to be p < 0.05.

## Results

### Chronic ethanol ingestion induces fibrosis in rat kidney

**Fig 1:**
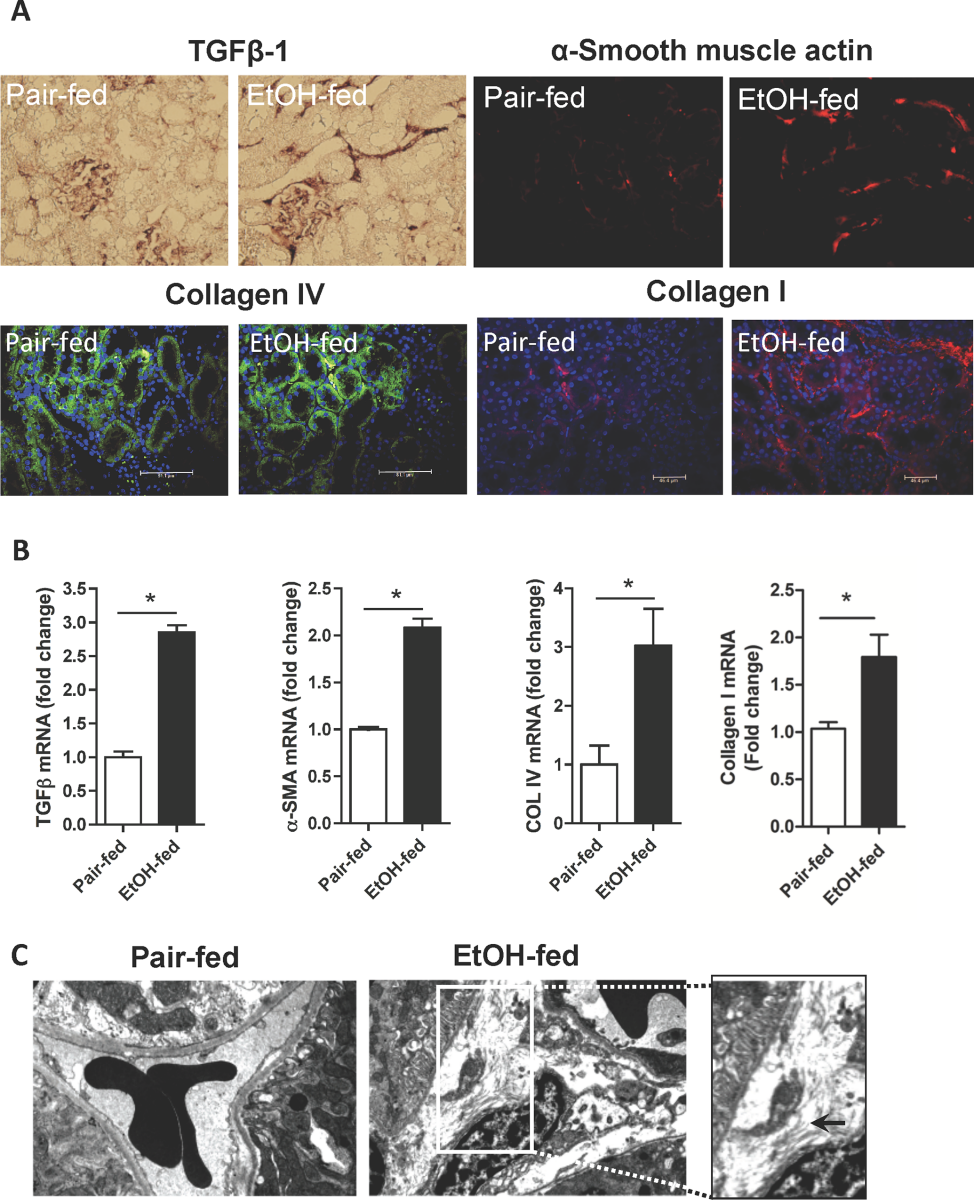
Chronic ethanol ingestion is fibrogenic in rat kidney. A) Fibrotic protein expression in kidney. Rats were fed the standard Lieber-deCarli liquid ethanol diet, or its isomaltose control, for 28 days before kidneys were excised, perfused with saline, fixed and sectioned as described in “Methods.” Sections were rehydrated and stained with anti-TGF-ß1, anti- α-smooth muscle actin, anti-collagen IV or anti-collagen 1 antibodies, and SP-streptavidin-conjugated secondary antibody for anti-TGF-ß1, or fluorescent secondary antibodies for the other sections as stated in “Methods.” TGF-ß sections were developed with horseradish peroxidase and DAB/metal chromogenic solution. B) mRNAs encoding fibrotic proteins were induced in kidneys of rats ingesting ethanol. Total RNA was isolated from ethanol and pair-fed kidneys from rats at the end of the feeding trial, and mRNA was quantified by SYBR Green one-step Reverse Transcription-PCR for the stated mRNAs and ribosomal 18S with the Bio-Rad MyiQ real-time PCR detection system. mRNA expression was normalized to 18S RNA content and 2^-ΔΔCT^ was used to calculate the fold changes. Data are expressed as mean±SEM (n = 4), and p<0.05 (*) was considered as significant. C) Ethanol induced extracellular fibril deposition in kidneys of rats chronically metabolizing ethanol. Kidneys of control pair-fed or ethanol fed rats were isolated, fixed, sectioned and prepared for electron microscopy as described in “Methods.” The micrograph from the control animal shows erythrocytes within a capillary separating three epithelial cells, in contrast to the disorganized milieu with extracellular fibrils found in the kidney of ethanol-fed rats. The inset to the right is an expanded micrograph with extracellular fibrils highlighted by an arrow.

The early steps in alcohol-induced liver damage are modeled in the Lieber-deCarli liquid ethanol diet where rats ingest a third of their caloric intake as ethanol and then are compared to control animals pair fed an isocaloric diet with isomaltose substituted for ethanol to equalize weight gain. Chronic ethanol ingestion in this model generates only mild liver inflammation by 4 weeks. Kidney, however, is exquisitely sensitive to exogenous ethanol, developing inflammation, loss of filtration, and acute kidney injury syndrome [10,28]. We determined whether inflammation progressed to renal fibrosis, despite the lack of fibrosis in liver, and so stained sections of kidney from rats ingesting a control and ethanol diet for fibrogenic proteins (Fig 1A). Chronic ethanol catabolism, in contrast to a control diet, induced expression in kidney of the fibrotic signaling cytokine TGF-ß1 (*top left*), α-smooth muscle actin of myofibroblasts (*top right*) and collagen IV (*bottom left*) and collagen I (*bottom right*). Quantitative analysis of mRNA for TGF-ß1 confirmed ethanol feeding increased transcripts for this cytokine, that the level of induction was significant, and that the increase in message was nearly 3 fold (Fig 1B). Alpha-smooth muscle actin protein was increased by ethanol ingestion, with a significant 2-fold increase in its mRNA. Similarly, collagen IV mRNA also was significantly increased by 3 fold and collagen I mRNA increased by 75% in the kidneys of ethanol fed rats. Electron micrographs (Fig 1C) showed an organized epithelium abutting an erythrocyte-containing vessel in control kidney, while specimens from ethanol-fed rats showed disorganized structures with accumulation of extracellular fibrils. We conclude kidney, in contrast to liver, responds to chronic ethanol metabolism with accumulation of fibrotic proteins and fibrils in the standard model of chronic ethanol ingestion.

### PTAFR is required for accumulation of fibrotic proteins in kidney during ethanol catabolism

**Fig 2:**
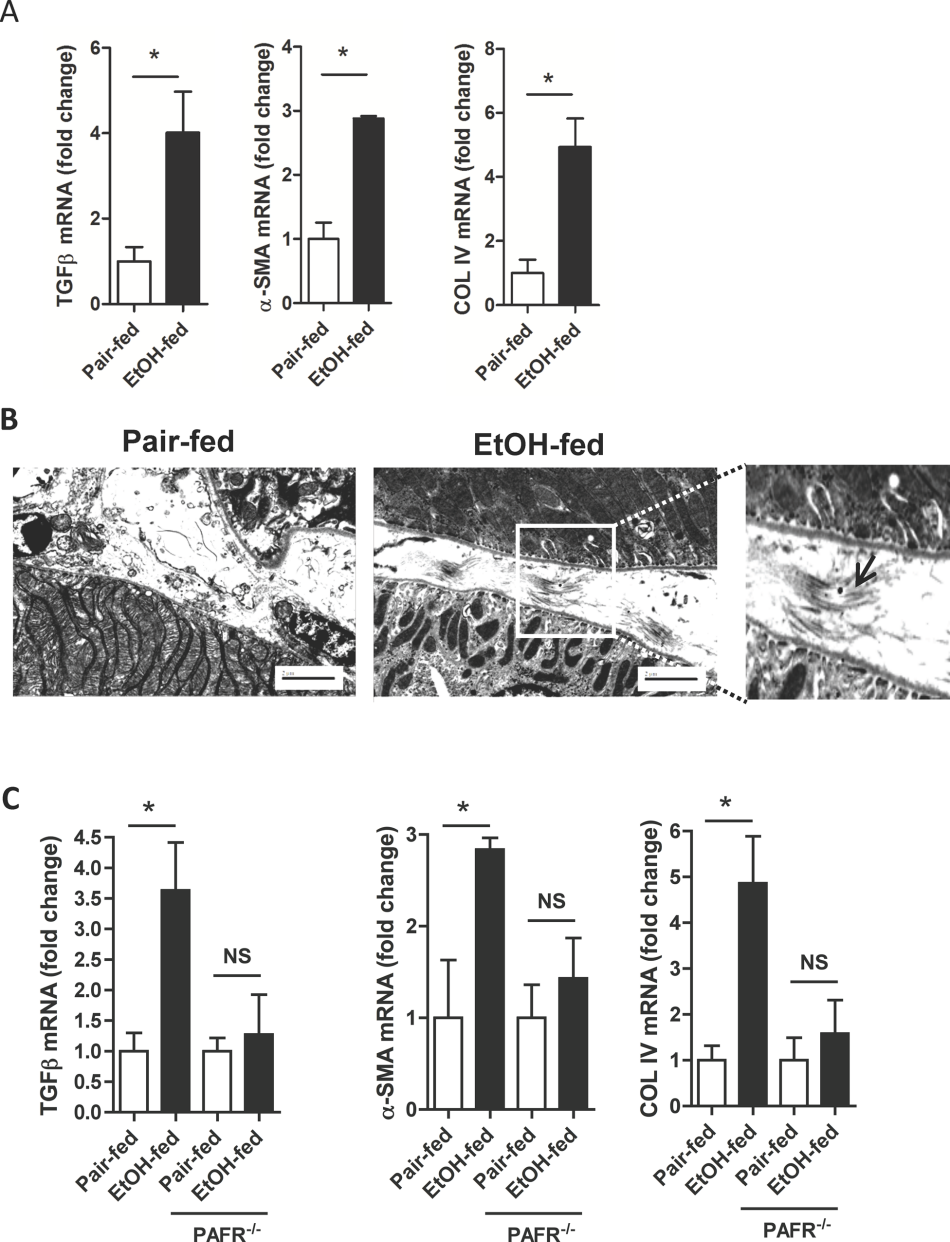
Renal fibrogenesis in mouse is PTAFR-dependent. A) Ethanol ingestion by mice induces mRNA encoding fibrotic proteins in kidney. mRNA was isolated from mice ingesting for 25 days a ramped ethanol diet or their controls was extracted and quantified relative to 18S RNA by real-time PCR as in Fig 1. Data are expressed as mean ± SEM (n = 4); *, p<0.05. B) Electron micrographs of kidney from control and ethanol-fed mice. Kidneys were recovered and imaged as in Fig 1, including 2 micron line inserts. Extracellular fibrils are present between epithelial cells in the ethanol-fed mice in the expanded micrograph on the right. C) Ethanol-induced accumulation of renal mRNA encoding fibrotic proteins requires PTAFR. mRNA was isolated and quantitated by qPCR as in panel A from control and ethanol-fed parental BL6 or *ptafr* ^-/-^ mice. The data is presented as mean ± SEM (n = 4); *, p<0.05.

Chronic ethanol ingestion causes kidney inflammation in mice chronically ingesting ethanol [28], as it does in rats [10], although the dietary protocol must be modified to overcome early aversion of mice to ethanol. We determined whether mouse kidney was affected by ethanol ingestion like rat kidneys to find excessive accumulation of both protein and message for TGF-ß, alpha-smooth muscle actin, and collagen IV in murine kidney (Fig 2A). The level of induction of mRNA for these fibrogenic proteins differed only slightly between the two animal models of chronic ethanol ingestion with mRNA accumulation in mice being somewhat more robust than in rat kidney. Also in concordance with the standard rat model, extracellular fibers were deposited in the kidneys of ethanol-fed mice (Fig 2B). We conclude ethanol metabolism induces fibrosis in both rat and mouse kidney, with the latter model allowing genetic intervention to determine essential components of kidney fibrosis in response to chronic ethanol ingestion.

Ethanol catabolism by CYP2E1 generates ROS that oxidize and fragment membrane polyunsaturated phospholipids to ligands for the PTAFR receptor for inflammatory phospholipids. Accordingly, genetic deletion of this receptor fully abolishes autocrine PTAFR ligand formation, neutrophil infiltration, and development of acute kidney damage and dysfunction [28]. We determined whether fibrosis also was suppressed in *ptafr* ^-/-^ mice ingesting ethanol compared to wild-type BL6 mice. We found the amount of mRNAs encoding TGF-ß, alpha-smooth muscle actin, and collagen type IV in the kidneys of mice that lack PTAFR did not increase in response to ethanol feeding (Fig 2C) as occurred in kidneys of wild-type mice metabolizing ethanol. We deduce that either local PTAFR signaling within kidney stimulates expression of these genes during ethanol catabolism, or that the PTAFR-induced inflammatory response is a critical intermediate between this receptor and induction of pro-fibrotic proteins.

### Ethanol ingestion induces Sonic hedgehog expression in kidney through PTAFR signaling

**Fig 3:**
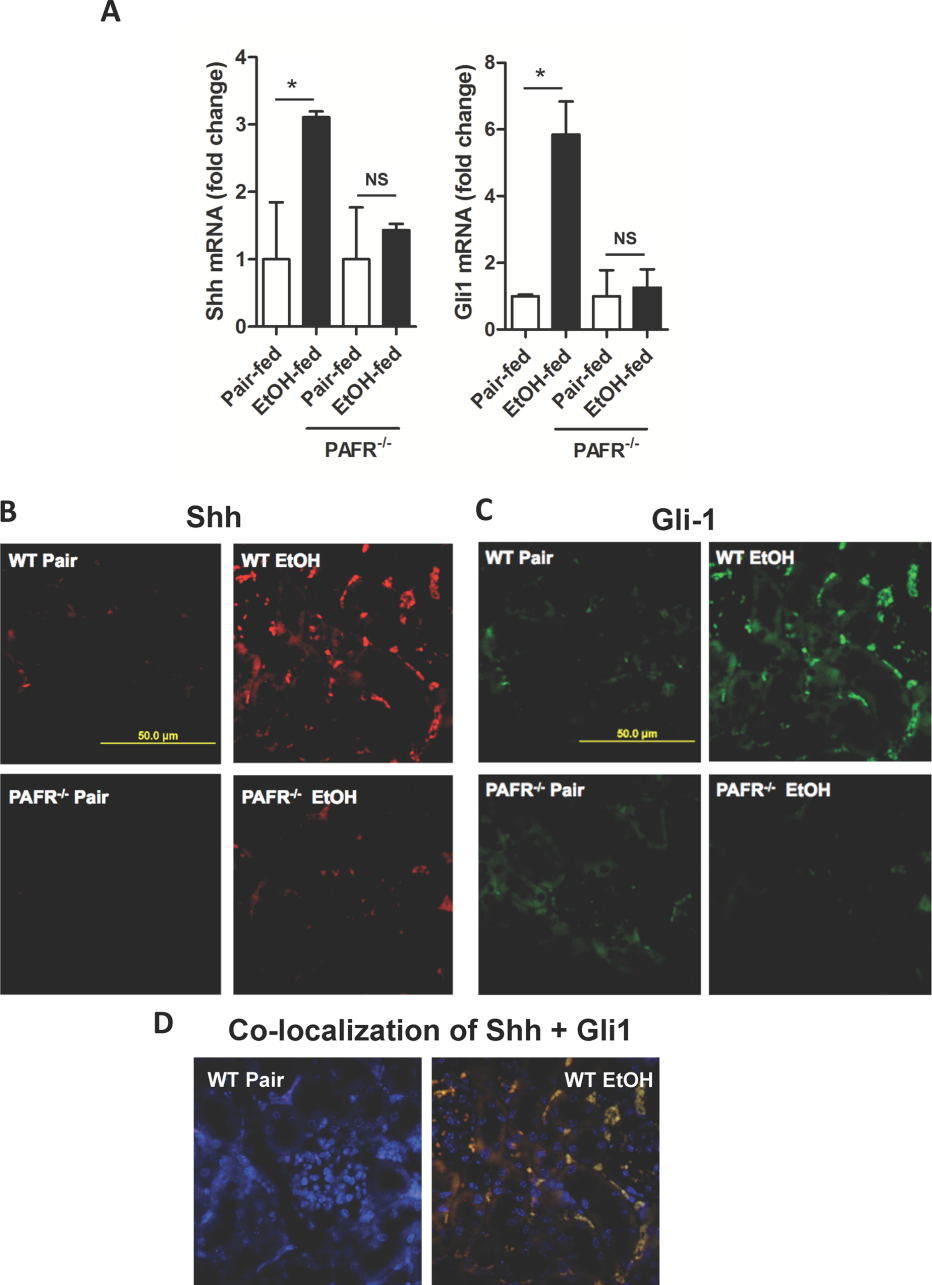
Ethanol induces expression of Sonic hedgehog and its Gli1 transcription factor in murine kidney. A) Ethanol-induced accumulation of Sonic hedgehog and Gli1 mRNA is dependent on PTAFR. Kidneys of wild-type BL6 or *ptafr* ^-/-^ mice ingesting ethanol or control diets were harvested and mRNA was extracted for qPCR quantitation and analyzed as in Fig 1. SEM (n = 4); *, p<0.05. B) Shh induction by chronic ethanol ingestion is dependent on PTAFR. Kidneys of wild-type BL6 or *ptafr* ^-/-^ mice fed ethanol or their pair-fed controls were harvested and stained by fluorescent immunohistochemistry for Shh and Fluor 568 secondary antibody. C) Gli1 induction by chronic ethanol ingesting is dependent on PTAFR. Sections of kidneys of mice with the stated genotype and diet were stained with anti-Gli1 and Fluor 488-conjugated secondary antibody as in the preceding panel. D) Shh and Gli1 co-localize in the kidneys of ethanol-fed mice. Image overlay of Panels B and C show Shh and Gli1 co-localize, and that the doubly positive cells are positioned to surround vascular lumens as shown by inclusion of a panel reporting DAPI (fluorescing blue) counterstained nuclei.

The hedgehog signaling pathway induces fibrosis in acutely damaged kidney [25,31], and we find mRNA for Sonic hedgehog (Shh) also was significantly induced in the kidney when the insult was chronic ethanol metabolism (Fig 3A). As with inflammation [28], genetic abolition of *ptafr* abolished the increase in Shh mRNA. Similarly, the transcription factor Gli1, which is induced by hedgehog signaling [22], also was induced in the kidneys of ethanol-fed mice, and loss of PTAFR signaling abolished the increase in Gli1 mRNA. Fate mapping shows kidney pericytes are the precursors of kidney myofibroblasts when injury is acutely induced by unilateral ureteral obstruction [25,31], and that this response is through Shh signaling [32]. Fluorescent immunohistochemistry showed expression of both Shh (Fig 3B) and Gli1 (Fig 3C) proteins were increased in the kidneys of ethanol-fed mice and that the two proteins were specifically associated with cells surrounding microvessels. Overlaying the images of fluorescent anti-Shh and anti-Gli1 showed these two proteins were extensively co-localized (Fig 3D). These data show ethanol induces Shh along with Gli1 in the kidney during chronic ethanol feeding and, like the ethanol-induced inflammatory response, that Gli1 and Shh induction require PTAFR.

### Ethanol feeding induces Gli1 transcription in kidney pericytes

**Fig 4:**
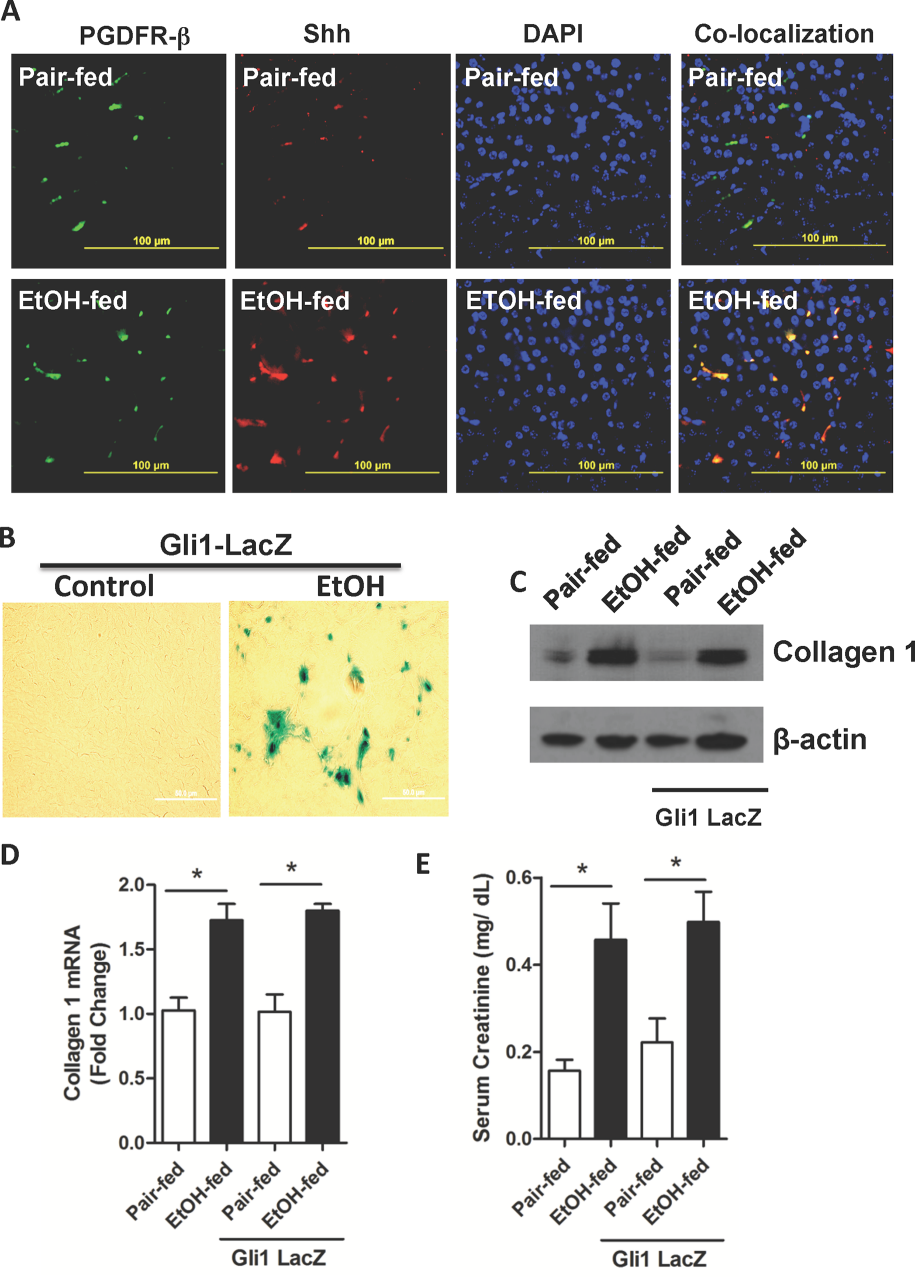
Ethanol ingestion induces Shh and Gli1 production and accumulation in renal pericytes. A) Renal Shh and PDGFR-ß1 co-localize after ethanol ingestion. Fluorescent immunohistochemistry of PDGFR-ß1, Shh, or DAPI nuclear counterstain of renal sections from mice ingesting a ramped ethanol diet or pair-fed a control diet. Image overlay in the right panel of green PDGFR-β and red Shh shows co-expression in yellow. B) Ethanol induces Gli1 transcription. ß-Galactosidase expression in kidney under control of the *gli1* locus in kidneys of pair-fed control and ethanol-fed heterozygotic Gli1^tm2Alj^/J reporter mice was assessed by hydrolysis of fluorogenic LacZ as described in “Methods.” C) Collagen accumulates in the kidneys of wild-type and Gli1 reporter mice. Immunoblots of collagen 1 protein in kidney lysates of wild-type or *gli1-LacZ* mice pair-fed a control diet or fed the ramped ethanol diet for 25 days. D) Collagen message is increased in Gli1 reporter mice. Collagen 1 mRNA quantified by qPCR relative to ribosomal 18s RNA in the kidneys of wild-type or reporter mice. SEM (n = 4), * p<0.05 E) Ethanol feeding damages renal function in Gli1 reporter mice. Circulating creatinine concentrations in *gli1-LacZ* or wild-type mice ingesting ethanol or a paired control diet with isomaltose isocalorically substituting for ethanol. SEM (n = 4). *, p<0.05

We determined whether Shh induced in response to chronic ethanol ingestion was a product of fibrotic kidney pericytes [24–27], and then whether Gli1 is induced in these cells in ethanol-fed animals. We localized the renal pericyte marker PDGFR-ß1 [33,34] by fluorescent immunohistochemistry, which was not detectably affected by the diet (Fig 4A). Shh, stimulated in kidney by chronic ethanol ingestion, co-localized with this PDGFR-ß1 with only a few cells identified as expressing Shh, but not PDGFR-ß1. We determined whether Gli1 expression was transcriptionally controlled using heterozygotic ß-galactosidase reporter *gli1-LacZ* mice that still retain Gli1 function from the wild-type allele. Fluorogenic X-Gal hydrolysis by ß-galactosidase showed transcription of the *gli1* gene was induced in response to ethanol feeding, with the majority of the cells actively transcribing ß-galactosidase being localized in a perivascular position (Fig 4B). We next confirmed that ethanol induced renal fibrosis in *gli1-LacZ* mice as well as wild-type animals to find that accumulation of fibrillar collagen 1 in response to this stress was identical to its induction by ethanol catabolism in wild-type mice (Fig 4C). Chronic ethanol consumption by these *gli1-LacZ* mice also increased mRNA for collagen I that was indistinguishable from the response of wild-type animals (Fig 4D). In addition, heterozygotic mice expressing ß-galactosidase under the control of the Gli1 promoter behaved as wild type mice with functionally compromised kidneys that allowed creatinine to accumulate in the circulation during ethanol feeding in a fashion identical to wild-type animals (Fig 4E). The *gli1-LacZ* reporter mice therefore phenocopy wild-type mice and accurately report *gli1* induction by ethanol.

### Ethanol induces Indian hedgehog expression in kidney tubules accompanied by loss to urine

**Fig 5:**
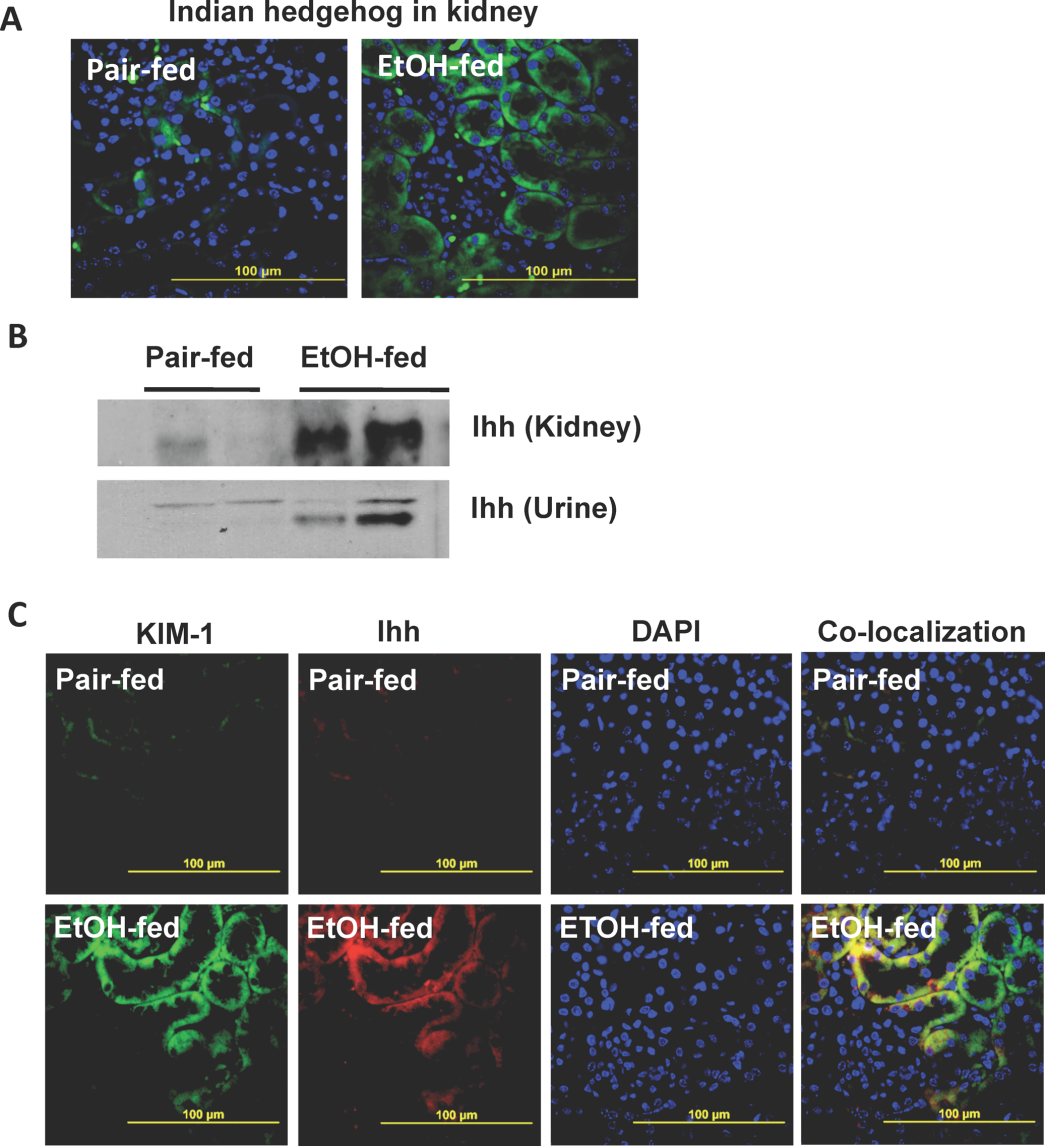
Chronic ethanol feeding allows Indian hedgehog escape to urine. A) Chronic ethanol ingestion induces Ihh protein expression in kidney. Ihh expression in kidney was determined by fluorescent immunohistochemistry in mice ingesting a control (*left*) or ethanol (*right*) diet. Nuclei were counterstained blue with DAPI mounting media. B) Ihh is present in the urine of ethanol-fed mice. Urine from either pair-fed control animals or that from mice fed a ramped Lieber-deCarli ethanol diet was collected at the end of the feeding trial. Urinary proteins and kidney homogenates were resolved by SDS-PAGE and immunoblotted for Ihh as described in “Methods.” C) Ihh and KIM-1 co-localize in the kidneys of ethanol-fed mice. Fluorescent immunohistochemistry of KIM-1, Ihh, or DAPI nuclear counterstain of renal sections from mice ingesting a ramped ethanol diet or pair-fed a control diet. Image overlay in the right panel of green KIM-1 and red Ihh shows co-expression in yellow.

We determined whether Shh was the only Ptch ligand induced by ethanol consumption to find that Indian hedgehog (Ihh) also was significantly induced in the kidneys ethanol-fed animals relative to mice ingesting a control diet (Fig 5A). The pattern of Ihh expression, however, was distinct from that of Shh (compare Fig 3D with Fig 4A). Western blotting confirmed that ethanol feeding induced Ihh protein within murine kidney (Fig 5B). Notably, western blotting additionally showed that a portion of this Ihh appeared in the urine of mice catabolizing ethanol. The protein kidney injury molecule-1 (KIM-1) is very strongly induced in tubular epithelium in response to renal injury [35], so we used fluorescent immunohistochemistry to determine whether Ihh was co-expressed with KIM-1. We observed equally strong induction of KIM-1 and Ihh as a consequence of ethanol feeding, and registering the two fluorescent micrographs showed complete, and clear, induction of Ihh along with KIM-1 in renal tubules of mice catabolizing ethanol (Fig 5C).

### Ethanol induction of the hedgehog pathway requires myeloperoxidase

**Fig 6:**
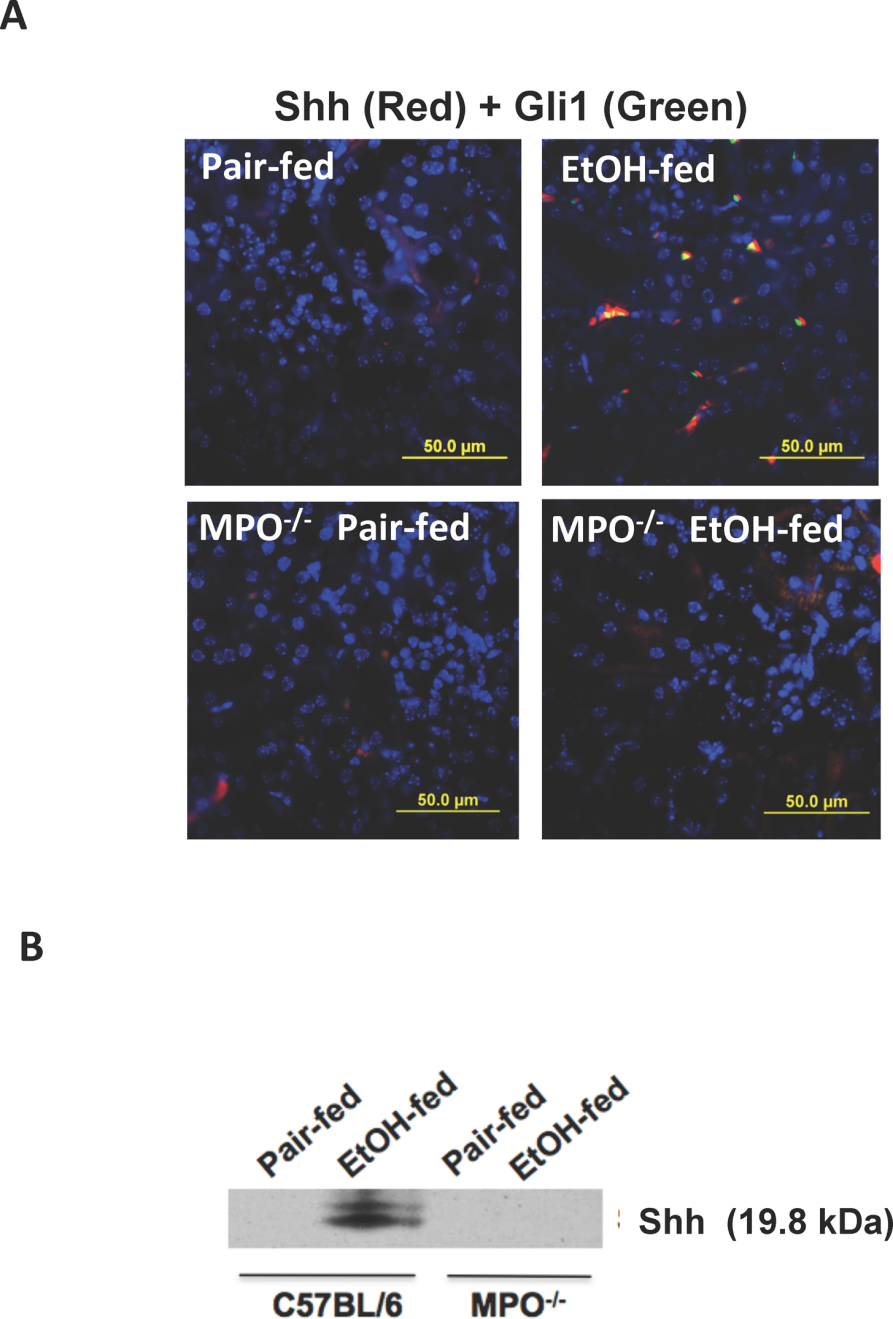
Myeloperoxidase is required for ethanol-induced expression and release of Shh. A) Shh accumulation in kidneys of ethanol-fed mice depends on myeloperoxidase. Kidneys of wild-type BL6 or *mpo* ^-/-^ mice chronically ingesting ethanol or a control diet were excised, fixed and fluorescently stained for Shh, Gli1, or nuclei with DAPI. The resulting images were overlaid to generate yellow co-localized proteins as in Fig 3. B) MPO is required for Shh accumulation in urine. Urine of wild-type and *mpo* ^-/-^ mice collected at the end of the feeding trail was resolved and immunoblotted for urinary Shh as in Fig 5.

Myeloperoxidase is an essential element of ethanol-induced kidney damage and dysfunction that distinguishes events that require leukocytic inflammatory infiltration from damage arising from local events that depend only on internal renal processes [28]. We found neither Shh nor Gli1 were induced during chronic ethanol ingestion in the kidneys of *mpo* ^-/-^ mice (Fig 6A). Surprisingly, since Shh was localized to a perivascular location not adjacent to tubular lumens, urine produced by the ethanol-fed wild-type mice contained Shh (Fig 6B). Loss of the myeloperoxidase gene abolished Shh release to urine. These data show that urinary Shh is a function of neutrophil stimulation by chronic ethanol metabolism, and that the presence of this mediator in urine correlated to its production in kidney.

### Urinary Shh marks acute kidney injury

**Fig 7:**
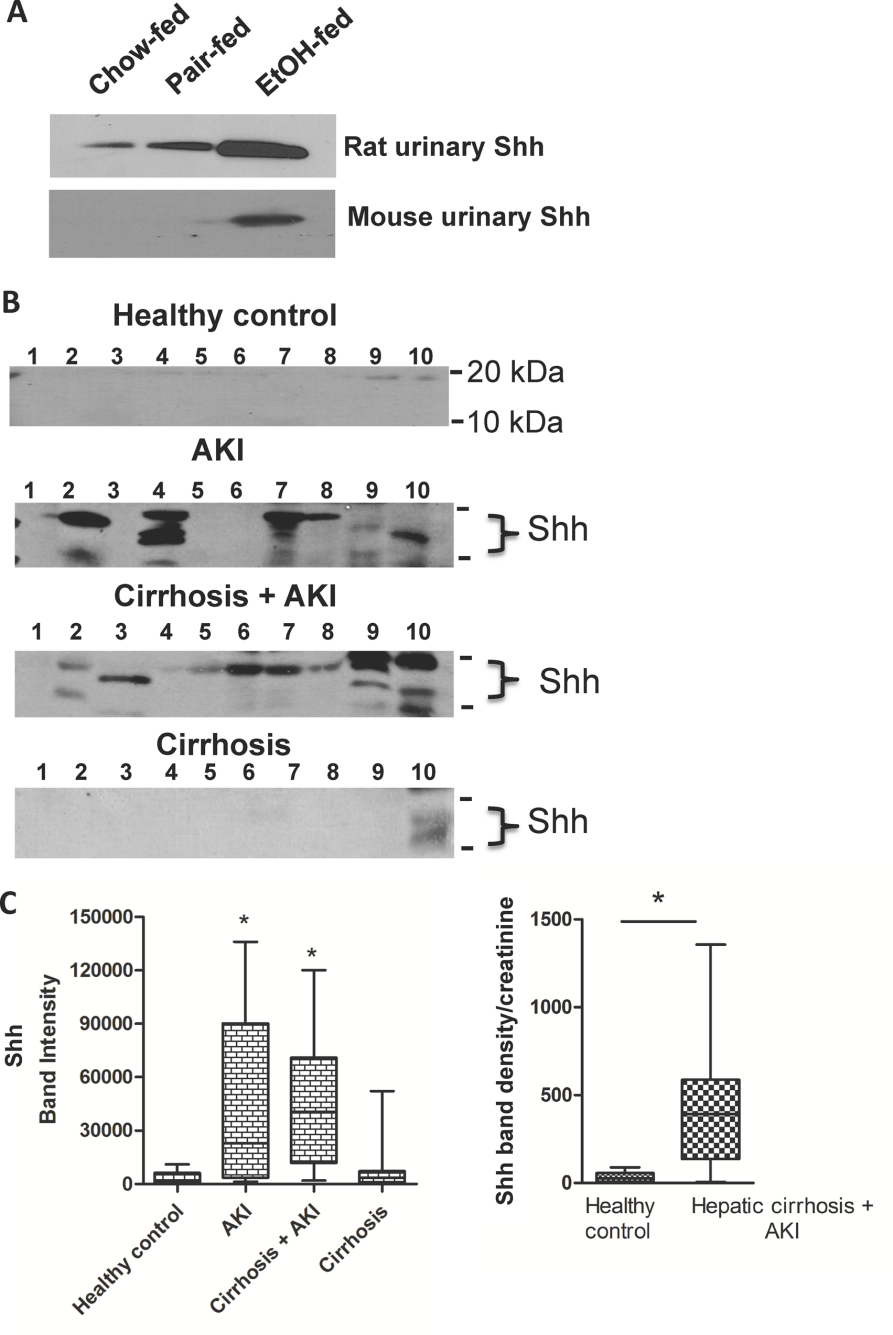
Shh is released to human urine during acute kidney injury independent of cirrhotic liver damage. A) Chronic ethanol ingestion in rodent models enhances Shh release into urine. Urine was collected from rats (*top*) or mice (*bottom*) ingesting ethanol for 28 or 25 days, respectively, and immunoblotted for Shh as in Figs 5 and 6. B) Humans with acute kidney injury release Shh into to urine. Spot urine samples from normal health controls or hospitalized patients diagnosed with cirrhosis, acute kidney injury (AKI), or with both cirrhosis and acute kidney injury were western blotted for Shh as in the preceding panel. The position of molecular size markers (10 kDa, 20 kDa) are depicted at the right side of the gels. Active Shh as a lipidated protein migrates between these size markers. C) Shh in patient urine correlates to acute kidney injury. *Left*. Statistical analysis of digitized immunoblots of urinary Shh in panel B among healthy controls, patients diagnosed with acute kidney injury, liver cirrhosis or a combination of the two diseases. N = 10 * p<0.05. *Right*. Shh density in immunoblots normalized by urine production using the Shh/creatinine ratio (S2 Table) and plotted as a bar and whisker graph showing the median and quartiles. N = 9 * p<0.05.

We determined whether Shh was shed to urine in the original rat model of chronic ethanol ingestion to find that Shh indeed was present in rat urine, but that in contrast to mice, urine from the pair-fed control rats contained detectable amounts of Shh (Fig 7A). We also found Shh was present in the urine of rats ingesting regular chow diets, so a low level of Shh release is constitutive in rats, but not in mice. We next determined whether Shh was present in human urine, and, if so, whether this mediator would be prevalent in patients with acute kidney disease. We also wished to know whether urinary Shh correlated to kidney or liver damage in humans. We found little Shh in urines of normal donors, but that Shh was variably present in hospitalized patients with acute kidney injury (Fig 7B). Urinary Shh was similarly prevalent in patients with combined cirrhosis and acute kidney injury from several diagnoses (S1 Table), but was absent in patients diagnosed only with cirrhosis. Comparison of the intensity of these blots shows patients with the syndrome of acute kidney injury, with or without the complication of cirrhosis, significantly differ in the amount of Shh in the urine relative to healthy control individuals (Fig 7C). Urinary Shh was not simply the result of altered urine production, assessed by accumulation of constantly secreted creatinine, since the Shh/urinary creatinine ratio also was significantly altered even in patients with combined AKI and cirrhosis (Fig 7C, S2 Table). We conclude, based on results with the pre-clinical murine model that urinary Shh closely correlates to expression of Shh within kidney, and so we conclude that urinary Shh also correlates to kidney injury in humans, and we conclude that urinary Shh does not correlate to liver fibrosis.

## Discussion

Ethanol is the most common xenobiotic encountered by Americans with its use estimated, although this is difficult to accurately assess, to be perhaps 8% of the American adult population [1]. However using the American Psychiatric Association’s new definition of Alcohol Use Disorder, the prevalence over an individual’s lifetime approaches 30% [36]. For most individuals, this exposure to dietary ethanol is not problematic, but a large proportion of individuals—perhaps a third of heavy alcohol users—will develop liver inflammation [1,37,38]. This steatohepatitis may progress to hepatic cirrhosis, fibrosis, and ultimately cancer, but steatohepatitis alone associates with significant mortality [3]. Thus, mortality of patients hospitalized with severe alcoholic hepatitis tightly associates with the sudden onset of acute kidney injury [3], and so is not the result of liver damage itself since patients with only liver damage were at little to no risk. This rapid decompensation of renal function indicates kidney itself may be compromised in the at-risk alcoholic population, although the insensitive and delayed [2] markers Blood Urea Nitrogen and creatinine fail to detect this. We currently lack a molecular understanding of the effect that chronic ethanol catabolism has on kidney, and so lack accurate, predictive measures of kidney dysfunction relevant to ethanol-induced damage in patients at risk of sudden loss of kidney function.

The early steps in alcohol-induced liver damage have long [39,40] been modeled in rats, but now also mice, fed an ethanol-containing diet that are compared to control animals pair fed an isocaloric control diet. The disease induced by ethanol ingestion in rodents, however, generates only mild liver inflammation that fails to progress to liver fibrosis and cirrhosis [41], as it does in humans [42]. Only by imposing binge ethanol exposure by gavage during chronic ethanol feeding can mild hepatic fibrosis be induced through adipocyte action [43]. In contrast, kidney is profoundly affected by chronic ethanol catabolism [10,28]. Kidney expresses between 5 and 10% of the total body content of CYP2E1 [7,8,10,44,45] and, as in liver, this ethanol catabolic enzyme accumulates in response to ethanol exposure [28]. CYP2E1 has a propensity to form the reactive oxygen specie (ROS) superoxide (O_2_^•-^), and ethanol oxidation by CYP2E1 generates sufficient ROS to truncate cellular phospholipids to PTAFR agonists [28]. Kidney abundantly expresses PTAFR that is intimately involved in kidney filtration [46], injury [47], and fibrosis [48]. Genetic ablation of PTAFR abolishes dietary ethanol-induced oxidation of kidney tissue, formation of PTAFR ligands in kidney, renal inflammation, and development of acute kidney injury [28]. Conversely, pharmacologic inhibition of the enzyme that selectively degrades PAF, the PAF acetylhydrolase [49], enhances neutrophil adhesion to vascular [50] and urethral epithelial cells [51]. Kidney therefore can expand the initial oxidative insult from ethanol oxidation in PTAFR-dependent in ways that liver, which generally lacks PTAFR, cannot. Ethanol catabolism, then, can have a direct, liver-independent effect on kidney structure and function.

Acute inflammation either resolves or progresses to chronic kidney injury where functional nephrons are lost and replaced by extracellular matrix. We find extensive neutrophil influx into kidney [28] with induction and accumulation of pro-fibrogenic proteins after just 25 days on the ethanol diet. Inflammation and fibrogenesis were temporally associated because renal inflammation only becomes evident in the last week of the ethanol diet [10,28] as fibrotic proteins accumulated. Inflammation was necessary to initiate renal fibrogenesis because deletion of *ptafr* prevented induction of TGF-ß, collagen IV, alpha-smooth muscle actin, Shh, and Gli1. These PTAFR deficient animals, however, continued to ingest and metabolize ethanol that constitutes up to a third of their caloric intake. Thus, it is not ROS formed by CYP2E1 catabolism of ethanol that were detrimental to kidney, but rather it is PTAFR-dependent oxidation and inflammation that were required for alcohol-induced fibrogenesis. Thus, neither liver nor kidney are extensively directly damaged by ethanol catabolism, but kidney, unlike liver, is susceptible to PTAFR-induced extension of the damage induced by ethanol catabolism. Instead, recruitment of inflammatory neutrophils into kidney, followed by in situ activation, was the essential element in kidney damage since loss of neutrophil MPO abolished inflammation [28] and fibrosis (*vide supra*).

Ethanol catabolism stimulated accumulation of Shh in Gli1-positive renal pericytes, which are the cell of origin for myofibroblasts in kidney [27,29], that attracts and instructs neutrophil migration into kidney [52]. Ihh also accumulated in response to the ethanol insult, but this occurred exclusively in kidney tubule cells. Potentially, production of a soluble hedgehog in one compartment could induce hedgehog accumulation in another environment. Both Shh and Ihh are soluble proteins that engage Smoothened signaling, but the promoters of the two hedgehogs differ with, for example, Ihh induction preceding Shh accumulation during radiation-induced liver fibrosis [53]. Ihh induction similarly might precede Shh induction in the kidneys during ethanol catabolism since Ihh co-localized in tubular cells along with CYP2E1-generated ROS [10,28]. Potentially, Shh signaling may begin the transition between inflammation and fibrosis through its anti-inflammatory effects stemming from IL-10 up-regulation [54] and MAPK pathway activation [55]. Alternatively, TGF-ß is an inducer of Gli1 transcription and Shh expression [56–58], and its expression may have a role in ethanol induction of fibrogenesis in kidney.

Acute kidney injury induces expression of Kidney Injury Molecule-1 on the surface of renal proximal tubules [59]. This molecule can then be shed to urine [35], creating a urinary marker of acute kidney injury [60]. Ihh also accumulated in kidney tubule cells along with KIM-1, and similarly was shed to urine produced by the kidneys of ethanol fed mice. Unexpectedly, we also found Shh was released to urine during ethanol feeding even though this mediator was localized to pericytes away from tubular lumens. In this case, like albumin that accounts for most of the proteinuria from ethanol-catabolizing kidneys, soluble Shh will escape to urine through compromised filtration. Ihh and Shh in urine correlated to their presence in kidney, which was confirmed by the loss of Shh in kidneys and urine of *ptafr* ^-/-^ and *mpo* ^-/-^ mice. Only tissues, such as kidney, with a neutrophilic inflammatory response to chronic ethanol ingestion, are possible sources of urinary Shh since genetic ablation of the *ptafr* and *mpo* loci prevented Shh release to urine. These results are consistent with, but are not proof of, kidney as the source of urinary Shh. Although kidney specific *ptafr* ^-/-^ animals are not available to confirm the site of urinary Shh production, this tight correlation suggests urinary hedgehog proteins are valid markers of ongoing hedgehog signaling and fibrogenesis in kidney, at least in pre-clinical models.

Shh shedding to urine extended to humans, where it associated with acute kidney injury. Shh was not normally present in the urine of healthy individuals, and the data show urinary Shh was not present in patients with cirrhosis. Shh, then, is a marker of renal injury independent of liver dysfunction and fibrosis, and so is not secondary to hepatorenal syndrome. In fact, patients 2 and 5 with acute kidney injury with cirrhosis were diagnosed with hepatorenal syndrome (S1 Table) but released only small amounts of Shh to urine. Potentially, Shh in human urine corresponds to renal fibrogenesis, and so may aid in defining individuals at risk of disruption of kidney structure prior to frank changes in filtration. Urinary Shh may thus identify individuals who might benefit from Gli1 inhibition. Whether urinary Shh and Ihh are selective markers of ethanol-induced kidney fibrogenesis, or are common to other forms of acute kidney injury is undefined, as is the utility of urinary hedgehog as prognostic markers of kidney disease.

## Supporting Information

S1 Table Etiology of acute kidney injury in patients with liver cirrhosis. Cirrhotic patients with acute kidney injury were from the listed syndromes and diseases. Patient number corresponds to the lane number of the urines immunoblotted in Fig 7C.
(DOCX)

S2 Table Western blot Shh band intensities, urinary creatinine values, and Shh to creatinine ratios. Band density of Shh was quantitized by in-gel staining with Li-Cor secondary antibody. This data, excepting the one record lacking urinary creatinine concentration, is plotted in Fig 7C. The patient number corresponds to those in S1 Table.
(DOCX)
