# Supplementary figures and images for: Inflammatory PAF Receptor Signaling Initiates Hedgehog Signaling and Kidney Fibrogenesis During Ethanol Consumption
Source: PLoS One. 2015 Dec 31;10(12):e0145691. doi: 10.1371/journal.pone.0145691 (PMC4697844; doi:10.1371/journal.pone.0145691)

**Table 1: Etiology of acute kidney injury in patients**

**with liver cirrhosis**


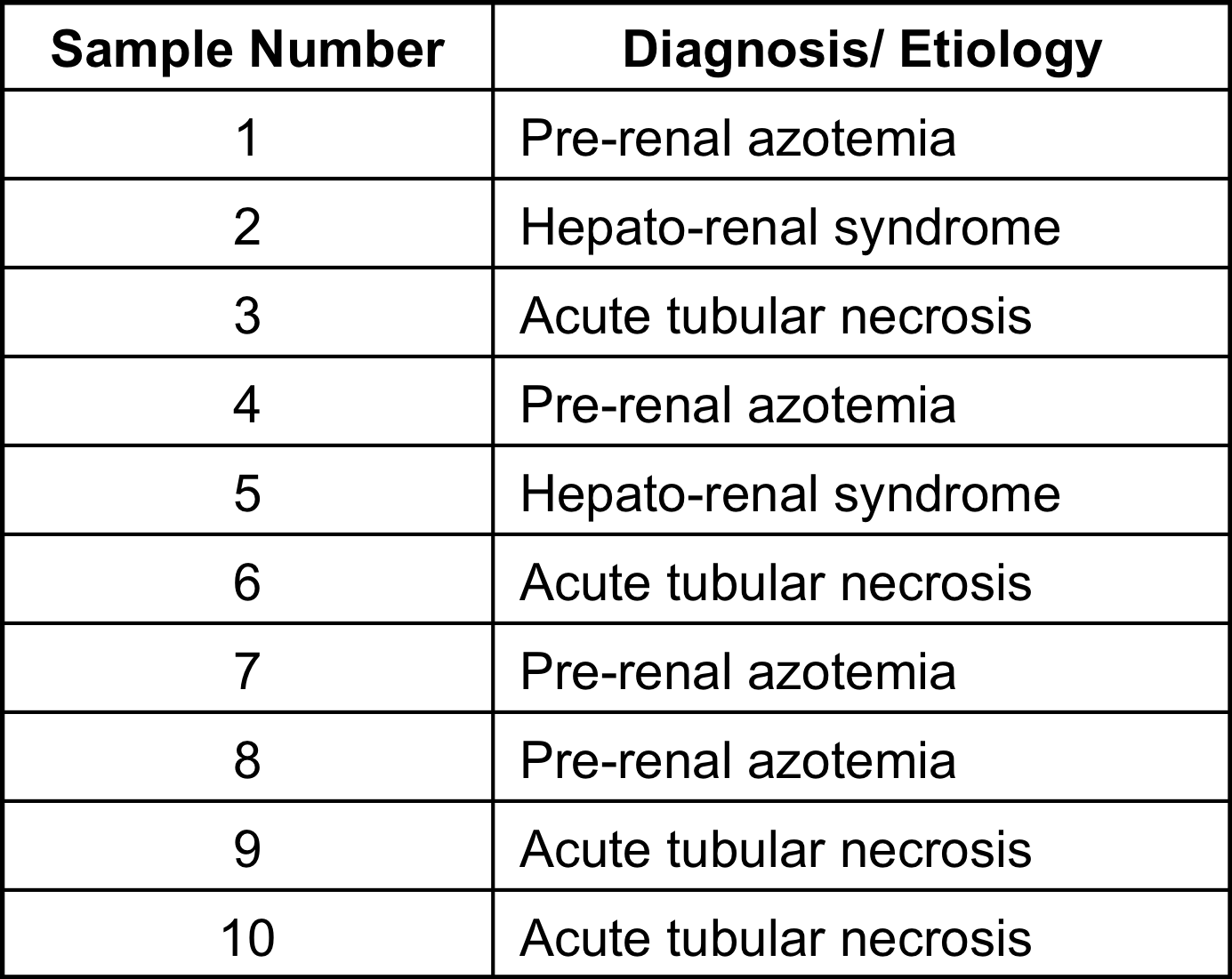

Supplement: S1 Table — Cirrhotic patients with acute kidney injury were from the listed syndromes and diseases. Patient number corresponds to the lane number of the urines immunoblotted in Fig 7C. (DOCX) [file pone.0145691.s001.docx]

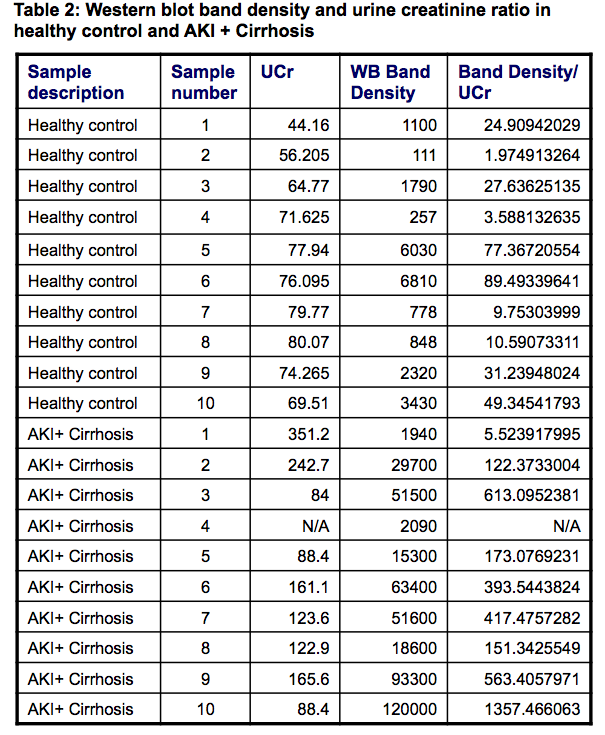

Supplement: S2 Table — Band density of Shh was quantitized by in-gel staining with Li-Cor secondary antibody. This data, excepting the one record lacking urinary creatinine concentration, is plotted in Fig 7C. The patient number corresponds to those in S1 Table. (DOCX) [file pone.0145691.s002.docx]
